# Automatic symptom name normalization in clinical records of traditional Chinese medicine

**DOI:** 10.1186/1471-2105-11-40

**Published:** 2010-01-20

**Authors:** Yaqiang Wang, Zhonghua Yu, Yongguang Jiang, Kaikuo Xu, Xia Chen

**Affiliations:** 1 Department of Computer Science, Sichuan University, Chengdu, Sichuan, PR China; 2 College of Preclinical Medicine, Chengdu University of TCM, Chengdu, Sichuan, PR China

## Abstract

**Background:**

In recent years, Data Mining technology has been applied more than ever before in the field of traditional Chinese medicine (TCM) to discover regularities from the experience accumulated in the past thousands of years in China. Electronic medical records (or clinical records) of TCM, containing larger amount of information than well-structured data of prescriptions extracted manually from TCM literature such as information related to medical treatment process, could be an important source for discovering valuable regularities of TCM. However, they are collected by TCM doctors on a day to day basis without the support of authoritative editorial board, and owing to different experience and background of TCM doctors, the same concept might be described in several different terms. Therefore, clinical records of TCM cannot be used directly to Data Mining and Knowledge Discovery. This paper focuses its attention on the phenomena of "one symptom with different names" and investigates a series of metrics for automatically normalizing symptom names in clinical records of TCM.

**Results:**

A series of extensive experiments were performed to validate the metrics proposed, and they have shown that the hybrid similarity metrics integrating literal similarity and remedy-based similarity are more accurate than the others which are based on literal similarity or remedy-based similarity alone, and the highest F-Measure (65.62%) of all the metrics is achieved by hybrid similarity metric VSM+TFIDF+SWD.

**Conclusions:**

Automatic symptom name normalization is an essential task for discovering knowledge from clinical data of TCM. The problem is introduced for the first time by this paper. The results have verified that the investigated metrics are reasonable and accurate, and the hybrid similarity metrics are much better than the metrics based on literal similarity or remedy-based similarity alone.

## Background

In recent years, Data Mining technology has been applied more than ever before in the field of TCM to discover regularities from the experience accumulated in the past thousands of years in China. The state of the art of Data Mining and Knowledge Discovery in TCM is described and several Data Mining methods in TCM are introduced in [1].

However, up to date all relevant work was based on well-structured data of prescriptions extracted manually from TCM literature. For example in [2], based on the prescriptions collected manually and organized into two datasets, a series of algorithms were developed and validated for discovering multi-dimensional major medicines. In [3] an algorithm was proposed to mine the associations between different items of medicine from a well-structured dataset which was also manually extracted from TCM literature by TCM experts. Collecting data in such a way is time-consuming, tedious and infeasible, and it is impossible to provide enough volume of data for inducing sufficiently reliable knowledge. Moreover, TCM literature does not provide enough information on the dynamic process of medical treatment which could become an important source for discovering valuable regularities in TCM.

Fortunately, electronic medical records (or clinical records) can compensate for the lack of the data collected from TCM literature. They contain large amount of information, especially the information of the whole medical treatment process. However, clinical records of TCM are made by TCM doctors on a day to day basis without the support of authoritative editorial board, and owing to different experience and background of TCM doctors, the same concept, especially symptoms, might be described in several different terms (78.41% (425/542) of the standard symptom names have more than one synonym (i.e. clinical symptom name) in our clinical datasets). Therefore, clinical records of TCM cannot be used directly to Data Mining and Knowledge Discovery.

This paper focuses its attention on the phenomena of "one symptom with different names" and develops a series of algorithms to normalize symptom names in clinical records of TCM. The core of the algorithms is measuring the similarity between the clinical symptom name to be normalized and all possible standard forms. Based on the similarity measurement, a clinical symptom name is normalized to its most similar standard form. If there is a tie in the most similar standard forms, one of them is chosen randomly as the standard form. Three types of similarity metrics are investigated for the purpose in this paper. The experimental evidences indicate that these instrumentalities are appropriate and accurate for automatically normalizing symptom names in clinical records of TCM.

## Methods

### Literal Similarity Metrics

Although symptoms are denominated by TCM doctors without the support of authoritative editorial board and a symptom might be described in several different names owing to different experience and background of TCM doctors, symptom names describing the same symptom usually have literal similarity due to the ideographic characteristics of Chinese. For example, both '' and '' mean head and they have the same ideographic character '' (Head). Both '' and '' mean that a person sweats in upper limb, and they also have the same ideographic characters '' (Upper Limb) and '' (Perspiration). Therefore, literal similarity metrics are considered to be used to measure the similarity between symptom names.

In spite of different experience and background of TCM doctors, symptoms are generally denominated with some loose conventions inherited historically and followed by most of TCM doctors. In general, a symptom name of TCM contains sequentially expressions of the affected body part, the disease property and the disease degree. For example, in the symptom name '' (Severe Headache) the affected body part is '' (Head), '' (Ache) is the disease property and '' (Severe) represents the disease degree. In '' (Throat Tickle) '' (Throat) is the affected body part with '' (Tickle) being the disease property. Among the components of a symptom name some may be missing such as in '' (Throat Tickle) the disease degree is absent. However, the component affected body part appears in most of symptom names (66.97% (363/542) of the standard symptom names and 70.10% (3130/4465) of the clinical symptom names contain the affected body part in our experimental data) and, moreover, it is usually the prefix when it appears in a symptom name (66.61% (361/542) of the standard symptom names and 55.83% (2493/4465) of the clinical symptom names start with the affected body part). Therefore, prefix of symptom names is considered to be an enhanced factor to determine the literal similarity.

According to the observations discussed above, four literal similarity metrics are used here for validating the feasibility, and Jaro-Winkler Distance is also used to demonstrate the effect of the symptom name prefix.

#### Jaro Distance Metric

Jaro Distance (JD) [4] is one of the most popular and basic literal similarity metrics, and here JD score is defined as follows:

Where *m *is the number of matching characters between a standard symptom name *s *and a clinical symptom name *s'*, *t *is the number of transpositions of the characters, i.e. the count of matching characters but in different order in *s *and *s' *[5], |*s*| and |*s'*| are the number of characters in *s *and *s' *respectively.

#### Jaro-Winkler Distance Metric

Jaro-Winkler Distance (JWD) [4] is extended from JD and adjusts the score of JD upwards for the symptom name pairs having common prefixes. JWD is introduced as follows:

Where *JD*(*s*, *s'*) is the JD score of a standard symptom name *s *and a clinical symptom name *s'*, *prefixLength *is the length of their common prefix, and *PREFIXSCALE *is a constant scaling factor for measuring how much the score is adjusted upwards for a symptom name pair having a common prefix (Here three is assigned to *PREFIXSCALE*).

#### Smith-Waterman Distance Metric

Smith-Waterman Distance (SWD) [6] is a dynamic programming algorithm, and it is guaranteed to find symptom name pairs which have the optimal local alignment with respect to a gap-scoring scheme and a scoring system including a substitution matrix. The substitution matrix *M *for comparing a symptom name pair is constructed as follows.

Where *sc*_*i *_is the *i*th character in a standard symptom name *s *and  is the *j*th character in a clinical symptom name *s'*, *m *is the length of *s *and *n *is the length of *s'*, *M*(*i*, *j*) is the similarity score between the substring *sc*_1_*sc*_2_...*sc*_*i *_of *s *and the substring  of *s'*, *ω *(*sc*_*i*_, ), *ω *(*sc*_*i*_, -) and *ω *(-, ) are the gap-scoring schemes described by [6] in detail.

#### Smith-Waterman-Gotoh Distance Metric

Smith-Waterman-Gotoh Distance (SWGD) [7] is an improved algorithm of SWD. It allows multiple-sized gaps, and speeds up to *O*(*MN*) instead of *O*(*M*^2^*N*) of SWD (where *M *and *N *are the lengths of a standard and a clinical symptom names respectively).

### Remedy-Based Similarity Metrics

According to the TCM theory, the same or similar symptoms are always treated by the same or similar groups of remedies (i.e. the corresponding remedies of the symptoms). For example, '' and '' are two similar symptom names representing throat pain in TCM, and they are both treated by the common remedies '' (Honeysuckle), '' (Chrysanthemum) and '' (Fructus Arctii). Therefore, the information about the corresponding remedies of a standard and a clinical symptom names is involved to determine whether they express the same symptom. Three remedy-based similarity metrics are proposed below to measure the similarity between a standard and a clinical symptom names using their corresponding remedies.

#### Set-Based Similarity Metric

The Set-Based similarity metric adopts Jaccard coefficient to measure the similarity between a standard and a clinical symptom names using their corresponding remedy sets. It is represented by the following formula.

Where *s *and *s' *are a standard and a clinical symptom names respectively, *R *and *R' *are their corresponding remedy sets, |*R *∪ *R'*| is the number of elements in the union of *R *and *R'*, and |*R *∩ *R'*| is the number of elements in the intersection of *R *and *R' *.

#### Vector-Space-Model-Based Similarity Metric

In TCM the remedy potency for curing different symptoms is not equivalent. Some remedies are often used to treat a symptom and seldom to treat the others. Appearance of such remedies is an important evidence to distinguish this symptom from the others. However, the Set-Based similarity metric does not measure and use the importance of remedies toward a particular symptom, presupposing that remedies are equivalent for all symptoms. To estimate the importance of a remedy toward a particular symptom, TF-IDF weighting scheme is involved as follows.

Let *s*_*i *_be a symptom name, *R*_*i *_be its corresponding remedy bag containing all the occurrences of remedies in the prescriptions with the symptom name *s*_*i*_, and *R *be the set of all remedies in TCM. For any *r*_*j *_∈ *R*, its weight *w*_*i*, *j *_for *s*_*i *_is defined as follows:

Where *f*_*i*, *j *_is the frequency of occurrence of *r*_*j *_in *R*_*i*_, |*R*| is the number of remedies in *R*, *df*_*j *_is the number of the symptom names whose corresponding remedy bags contain *r*_*j*_.

Thus a vector in multi-dimensional space is constructed naturally by the weighted remedies to describe every symptom name. For a standard symptom name *s*_*m *_and a clinical symptom name *s*_*n*_, if their corresponding remedy bags are *R*_*m *_and *R*_*n*_, the following vectors are used to describe *R*_*m *_and *R*_*n*_.

Then similarity between *s*_*m *_and *s*_*n *_can be measured by the cosine metric defined bellow.

#### SimRank-Based Similarity Metric

The Set-Based and Vector-Space-Model-Based similarity metrics presuppose the independence among the corresponding remedies. However, the hypothesis may be violated owing to the fact that some remedies are alternative i.e. they have the same or similar effects. For example, the remedies '' (Hawthorn) and '' (Endothelium Corneum Gigeriae Galli) have the same effect and they all can be used to treat the symptom '' (Anorexia). According to the intuition that "two objects are similar if they are related to similar objects" [8], an observation is derived that two symptom names may be same or similar if they have same or similar corresponding remedies and two remedies are similar (or they have similar curative effects) if they are used to treat same or similar symptoms. Following the observation and based on the SimRank algorithm [8], the mutually recursive computational process of *SimS *(the similarity of two symptom names) and *SimR *(the similarity of two remedy names) are described as follows.

(1) Initialize *SimS *and *SimR *as follows.

(2) Iteratively update *SimS *and *SimR *using the formulas below until the termination condition is met.

Where *k *represents the *k*th iteration and *k *≥ 1, *R *and *R' *are the corresponding remedy sets of symptom names *s *and *s' *respectively, |*R*| and |*R'*| are the sizes of *R *and *R'*, *r*_*i *_and  are the *i*th and the *j*th remedies in *R *and *R' *. Similarly, *S *and *S' *are the corresponding symptom name sets of *r *and *r' *(*S *and *S' *both contain standard symptom names as well as clinical symptom names), |*S*| and |*S'*| are the sizes of *S *and *S'*, *s*_*i *_and  are the *i*th and the *j*th symptom names in *S *and *S'*, *C *is called as 'confidence level' or 'decay factor' and it is a constant value between 0 and 1 (the signification and argument of *C *can refer to [8]). SimRank was introduced by [8] in detail. In this paper, when *k *equals 4 the iterative procedure is terminated.

### Hybrid Similarity Metrics

Both literal similarity metrics and remedy-based similarity metrics have their advantages respectively, but the disadvantages also exist. Literal similarity metrics cannot distinguish the symptom names which have high literal similarity but with different or even opposite meanings. Remedy-based similarity metrics can find similar symptom names which are cured by similar remedies, but they ignore the literal characteristics of symptom names.

Therefore, a hybrid strategy which integrates literal similarity and remedy-based similarity is investigated for making up for the disadvantages of each other. The strategy is drawn from the following observation.

Observation: Two ***s***ymptom names expressing the same symptom have the similar corresponding ***r***emedies, at the same time the ***s***ymptom names should be literally ***s***imilar (named *SRSS*).

According to the observation, the hybrid strategy (i.e. *SRSS*) is constructed as follows.

Where *s *and *s' *are a standard and a clinical symptom names respectively, *α *and *β *are the weights of *Sim*_*L*_(*s*, *s'*) and *Sim*_*RB*_(*s*, *s'*), *Sim*_*L*_(*s*, *s'*) denotes literal similarity which can be computed through any literal similarity metric discussed above, *Sim*_*RB*_(*s*, *s'*) expresses remedy-based similarity, and its definition can be chosen among all the remedy-based similarity metrics. Instantiation of *Sim*_*L*_(*s*, *s'*), *Sim*_*RB*_(*s*, *s'*) and their weights will result in a particular hybrid similarity metric.

## Results

### Experimental Datasets

Two datasets were used in the experiments. The first one was the 2008 SiJunZi Standard TCM Dataset (SJZSTCMD). It is a national standard dataset consisting of 4950 standard prescriptions with 947 distinct symptom names and 721 distinct remedies. The second one was a clinical record dataset (CRD) including 14857 clinical diagnosis records collected by TCM doctors during medical consultation. The clinical diagnosis records contain 4950 different clinical symptom names, each with a set of remedies prescribed by TCM doctors.

**Figure 1:**
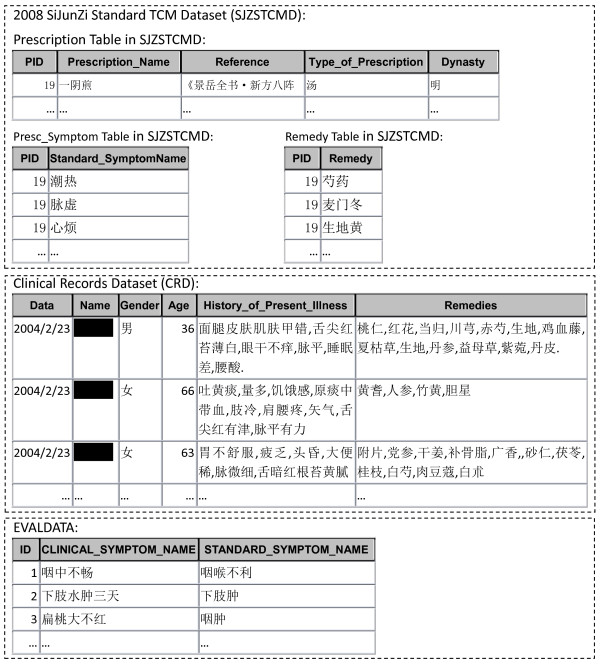
**Examples of datasets (SJZSTCMD, CRD, EVALDATA) used in experiments**.

In order to judge the output of our algorithms, the clinical symptom names were normalized in advance manually by TCM experts as the standard answers. Among the 4950 clinical symptom names, there are 485 clinical symptom names which do not have TCM meaning or could not be normalized to the standard symptom names. Thus the task of the experiments is to normalize the remaining 4465 clinical symptom names to one of the 947 standard symptom names. Examples of these primitive datasets are shown in figure 1.

### Data Pre-processing

The primitive CRD contains a lot of information needless for our algorithms such as format control characters ('-', '/', '=' and so forth), patient names. For simplicity of the subsequent normalizing, a step of data preprocessing was performed to filter out the needless information and extract clinical symptom names to be normalized and their corresponding remedies. The extracted clinical symptom names and their corresponding remedies were organized into an intermediate dataset which will become the input of our normalization algorithms.

### Evaluation Metrics

Precision, recall and F-Measure were used for evaluating the results, and they are defined as follows.

Where |*CNS*| is the number of clinical symptom names normalized correctly, |*NS*| is the number of clinical symptom names normalized, and |*CSN*| is the number of clinical symptom names to be normalized.

### Evaluation of Symptom Name Normalization

#### Literal Similarity Metrics

**Figure 2:**
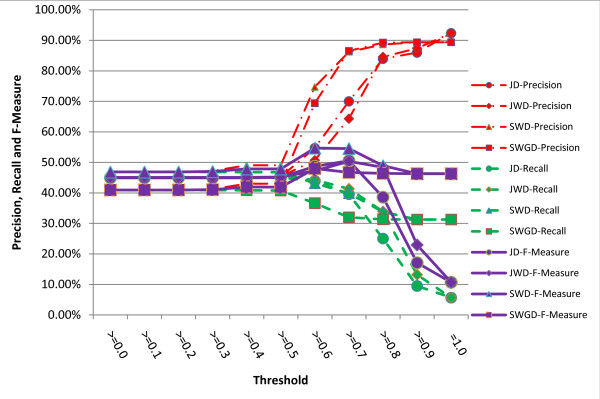
**Comparison of precisions, recalls and F-Measures obtained by different literal similarity metrics under different thresholds**.

Precisions, recalls and F-Measures of the literal similarity metrics under different thresholds are given in figure 2, which reveals that JWD is better than JD under almost all the threshold settings, and when the threshold is assigned to 0.8, F-Measure of JWD is about 9.84% higher than JD's. Such experimental result validates that prefix of symptom names indeed plays a key role in computing the literal similarity.

Figure 2 also demonstrates that the dynamic programming algorithm SWD has the best performance in terms of the precision, recall and F-Measure among all literal similarity metrics. Its highest F-Measure 54.72% is reached under precision 74.72%, recall 43.16% and the threshold 0.6. It is derived from figure 2 and the discussions above that the literal similarity metrics are reasonable to solve the problem of automatic symptom name normalization in clinical records of TCM.

#### Remedy-Based Similarity Metrics

**Figure 3:**
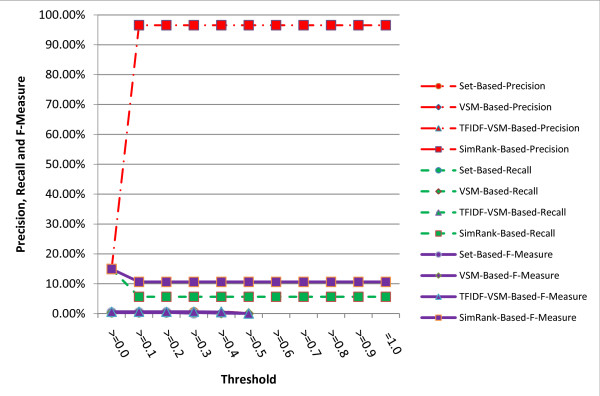
**Comparison of precisions, recalls and F-Measures obtained by different remedy-based similarity metrics under different thresholds**.

Precisions, recalls and F-Measures of the remedy-based similarity metrics under different thresholds are described in figure 3. The figure clearly shows that the SimRank-based similarity metric is the best one among all the three metrics regardless of the precision, recall or F-Measure, and its F-Measure is over ten times as high as the other two metrics. The SimRank-based similarity metric can achieve about 96.54% precision under threshold larger than 0.1. However, its recall and F-Measure are far beyond the literal similarity metrics. The empirical evidence proves that using corresponding remedies alone to normalize clinical symptom names is far worse than the literal similarity metrics.

#### Hybrid Similarity Metrics

**Figure 4:**
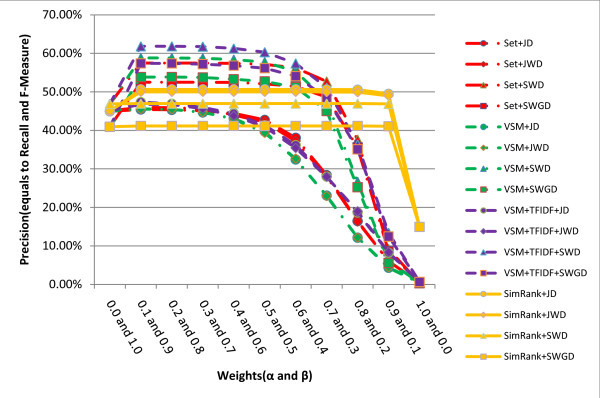
**Comparison of precisions, recalls and F-Measures obtained by different hybrid similarity metrics with different weights (*α *and *β*)**.

The hybrid similarity metrics weight and mix together the literal similarity and the remedy-based similarity in order to gain advantages of the two metric types. Precisions, recalls and F-Measures of the hybrid similarity metrics with different literal and remedy-based similarities and different weights *α *and *β *are shown in figure 4. It is represented that the SimRank-related hybrid similarity metrics are apparently the most stable methods when *α *and *β *are altered. The highest F-Measure of all the hybrid metrics is 61.84% (precision = 61.84%, recall = 61.84%) obtained by the hybrid similarity metric VSM+TFIDF+SWD when *α *= 0.1, *β *= 0.9, or *α *= 0.2, *β *= 0.8. Table 1 provides the best weights for every hybrid similarity metric.

**Table 1:** Weights (*α*, *β*) on making the optimized results of hybrid similarity metrics.

Hybrid Similarity Metrics	Weights
Set+JD	(0.1, 0.9)

Set+JWD	(0.1, 0.9)

Set+SWD	(0.1, 0.9); (0.2, 0.8); (0.3, 0.7)

Set+SWGD	(0.1, 0.9); (0.2, 0.8); (0.3, 0.7)

TFIDF+VSM+JD	(0.1, 0.9)

TFIDF+VSM+JWD	(0.1, 0.9)

TFIDF+VSM+SWD	(0.1, 0.9); (0.2, 0.8)

TFIDF+VSM+SWGD	(0.1, 0.9); (0.2, 0.8)

SimRank+JD	(0.1, 0.9); (0.2, 0.8); (0.3, 0.7); (0.4, 0.6); (0.5, 0.5); (0.6, 0.4)

SimRank+JWD	(0.1, 0.9); (0.2, 0.8); (0.3, 0.7); (0.4, 0.6); (0.5, 0.5); (0.6, 0.4)

SimRank+SWD	(0.1, 0.9); (0.2, 0.8); (0.3, 0.7); (0.4, 0.6); (0.5, 0.5); (0.6, 0.4); (0.7, 0.3); (0.8, 0.2)

SimRank+SWGD	(0.1, 0.9); (0.2, 0.8); (0.3, 0.7); (0.4, 0.6); (0.5, 0.5); (0.6, 0.4); (0.7, 0.3); (0.8, 0.2)

#### Comprehensive Evaluation

In order to investigate the metrics proposed more deeply, the literal similarity metrics are compared under different thresholds against their corresponding hybrid similarity metrics with the same weights (*α *= 0.1 and *β *= 0.9) which are the common best weights of the hybrid similarity metrics.

**Figure 5:**
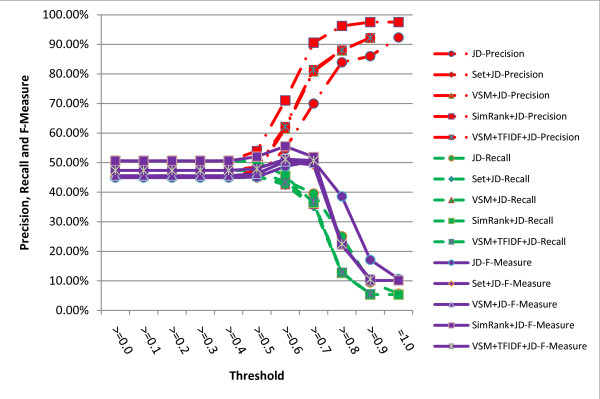
**Comparison of precisions, recalls and F-Measures obtained by JD and its corresponding hybrid similarity metrics under different thresholds**.

**Figure 6:**
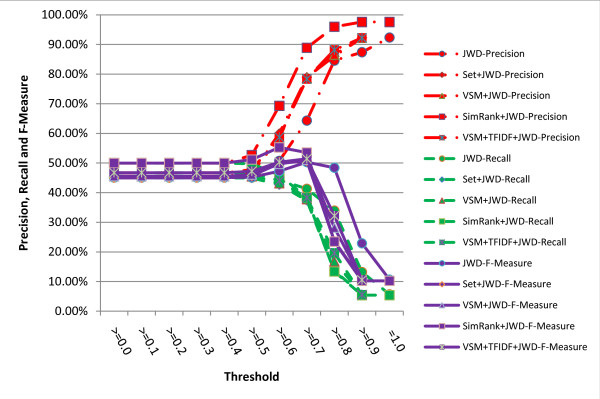
**Comparison of precisions, recalls and F-Measures obtained by JWD and its corresponding hybrid similarity metrics under different thresholds**.

**Figure 7:**
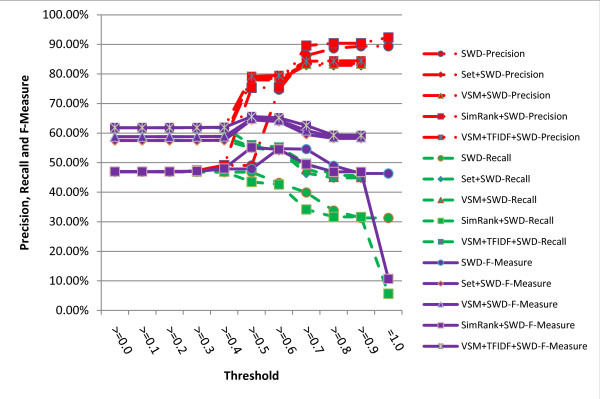
**Comparison of precisions, recalls and F-Measures obtained by SWD and its corresponding hybrid similarity metrics under different thresholds**.

**Figure 8:**
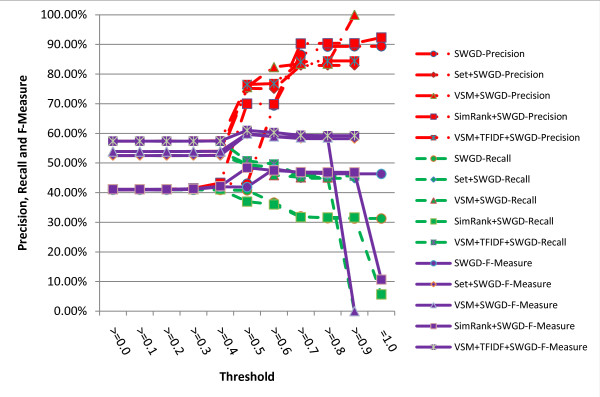
**Comparison of precisions, recalls and F-Measures obtained by SWGD and its corresponding hybrid similarity metrics under different thresholds**.

The results are shown in figures 5, 6, 7, 8. It turns out from the figures that precisions of the hybrid similarity metrics are higher than the literal similarity metrics in most cases, and the greatest difference under the same threshold between a hybrid similarity metric and a literal similarity metric is over 33.43% attained by VSM+TFIDF+SWGD and SWGD using a threshold of 0.5 (see figure 8). Figures 5 and 6 show that F-Measures of JD- and JWD-related hybrid similarity metrics are higher than JD and JWD's respectively when the threshold value is lower than 0.7. Figures 7 and 8 indicate that most of the hybrid similarity metrics' F-Measures are better than their corresponding literal similarity metrics' except SimRank+SWD and SimRank+SWGD's. The recalls of the hybrid similarity metrics are also better than their corresponding literal similarity metrics'.

The highest precision of all the metrics is 97.57% which is obtained by the hybrid similarity metric SimRank+JWD using a threshold of 0.9 (see figure 6). The highest recall (61.84%) is achieved by the hybrid similarity metric VSM+TFIDF+SWD with the threshold ranging from 0.0 to 0.4, and the hybrid similarity metric VSM+TFIDF+SWD attains the highest F-Measure 65.62% (precision = 79.18%, recall = 56.03%) when the threshold is set to 0.5.

In conclusion, the hybrid similarity metrics are more appropriate than the literal similarity metrics for solving the problem of automatic symptom name normalization in clinical records of TCM, and the corresponding remedies can be a useful factor for improving the effectiveness of normalization.

## Discussion

In clinical data of TCM non-standardization is a widely existing problem. Finding an appropriate approach to cope with this problem and to suit TCM theories can be a pivotal matter. In the fields of bioinformatics, linguistics, computer science and so forth, there are several approaches that can be used to cope with the problem of non-standardization. An unsupervised learning algorithm named PMI-IR was used to measure the similarity of pairs of words by Peter D. T [9], and it achieved satisfactory results. Several machine learning techniques, such as supervised learning, semi-supervised learning, unsupervised learning, and reinforcement learning, etc., have been used to resolve the problems of extracting synonymous gene and protein terms in biomedicine [10], and some record linkage methods and natural language processing approaches have also been used to solve name matching problems for finding the duplications [11-15]. All the above methods can be resolved into the literal similarity metric. In exploring the gene ontology [16], web services [17], natural language analysis [18] and so forth, the semantic similarity metric has been used. However, researchers rarely focus their attentions on the task of automatically normalizing terminology in TCM.

The experimental results performed in this paper indicate that the metrics for normalizing symptom names automatically in clinical records of TCM are appropriate, and they can provide more authentic clinical records for TCM researchers to improve the quality of study. At the same time, large amount of useful information, especially the information of the whole medical treatment process, would be further processed after the normalization. The deeper regularities in TCM would be also mined from the normalized clinical records through an array of proven Data Mining techniques. It has an overall positive effect on modernization of TCM.

The literal similarity metrics and the remedy-based similarity metrics have their advantages and disadvantages. Although the hybrid similarity metrics are more accurate than the others which are based on one of the evidences alone, only considering the literal similarity and the remedy-based similarity between TCM symptom names may be not enough. As the future work, some other significant characteristics would be included in order to improve the accuracy and effectiveness of the metrics.

## Conclusions

Automatic symptom name normalization is an essential task for discovering knowledge from clinical data of TCM. The problem is introduced for the first time by this paper. Based on the literal similarity and the remedy-based similarity, different metrics were investigated for this task and a series of experiments were performed to validate the metrics. The experimental results have proved that these metrics are reasonable and accurate, and the hybrid similarity metrics are better than the metrics which are based on literal similarity or remedy-based similarity alone.

## Authors' contributions

The theory was proposed by YW, and YW implemented the experiments and wrote the paper. ZY conceived the general ideas of automatic symptom name normalization and gave several suggestions to YW. YJ provided TCM theoretical directions and validated the results. And XC helped YW to implement the experiments and gave some helpful suggestions. Several suggestions about theories of computer science were suggested by KX. All authors read and approved the final manuscript.
